# Trends in Semiprofessional Scottish Football Players’ Acceleration and Deceleration Loads Across Playing Positions During Competitive Play and Training

**DOI:** 10.1155/tsm2/6295249

**Published:** 2026-09-09

**Authors:** Mark Farrell, Judith V. Lane, Prateek Rangra

**Affiliations:** ^1^ Division of Dietetics, Physiotherapy, Podiatry and Radiography, Queen Margaret University, Musselburgh, UK, qmu.ac.uk; ^2^ Forfar Athletic Football Club, Forfar, UK; ^3^ Musculoskeletal Physiotherapy Service, East Lothian Community Hospital Haddington, NHS Lothian, Haddington, UK, nhs.uk

## Abstract

**Background:**

High‐intensity accelerations (HIAccs) and high‐intensity decelerations (HIDecs) are critical to football performance, yet little is known about their demands in semiprofessional contexts where training frequency and resources differ from the professional game.

**Purpose:**

This study quantified HIAcc and HIDec loads during competitive match play and training across playing positions in semiprofessional Scottish football.

**Methods:**

Thirty‐three players were monitored over two competitive seasons (2021–2023) using 10‐Hz Global Positioning System (GPS) devices, generating 1333 individual observations. Linear mixed models assessed total distance, frequency and peak magnitude of HIAcc and HIDec at two intensity thresholds (3‐4 m/s^2^ and > 4 m/s^2^) across playing positions and session types (MD‐4, MD‐2 and match day).

**Results:**

Position (*p* < 0.001) and session type (*p* < 0.001) significantly influenced HIAcc and HIDec demands. Full‐backs recorded the highest deceleration loads, particularly > 4 m/s^2^, whilst wide midfielders accumulated greater acceleration distances than central players. MD‐4 sessions often exceeded match‐day acceleration values (106%–120%), whereas match days consistently imposed higher deceleration demands. These patterns diverged from those reported in professional cohorts.

**Conclusion:**

Semiprofessional players display distinct positional and session‐specific loading patterns compared with professional football. Tailored, position‐specific training and modified periodisation strategies appear necessary to align training with the physical demands of competition in this population.

## 1. Introduction

Recent years have witnessed growing interest in football load monitoring, which encompasses both internal (physiological) and external (physical) aspects [1]. Time‐motion analysis and Global Positioning Systems (GPS) provide detailed insights into players’ external loads during training and matches [2, 3]. Elite footballers typically cover total distances of 9000–14,000 m during a competitive match [4, 5]. Of this, 80%–90% is spent performing low‐intensity activity, whereas 10%–20% involves high‐intensity bouts such as high‐speed running (HSR) and sprinting [6]. Position‐specific differences are evident: Wide midfielders (WMs) and full‐backs (FBs) excel in HSR [7], whilst central defenders (CDs) engage in fewer sprints and high‐intensity activities [8, 9]. Central midfielders (MF), by contrast, often accumulate high volumes of moderate‐to‐high–speed transitional running due to their continuous involvement across both offensive and defensive phases [10], whilst forwards (FWs) frequently execute maximal sprint actions associated with penetrative or counter‐attacking movements [11]. These positional distinctions illustrate the heterogeneous locomotor requirements associated with different playing roles and emphasise the importance of interpreting external‐load profiles within a positional framework.

Players engage in both acceleration and deceleration activities in competitive matches, constituting approximately 18% of the total distance covered [12], with HIDec (> 3 m/s^2^) occurring more frequently than HIAcc (> 3 m/s^2^) per game [13]. Research also reveals distinctive patterns amongst player positions. MF exhibit a higher frequency of accelerations and decelerations at lower thresholds than CD, FB, WM and FW players [14]. In contrast, FB and WM show superior acceleration and deceleration densities (total distance and frequency) than MF and CD [14]. Moreover, Tierney et al. [15] suggested that WM achieves greater HIDec than any position and exhibits the most significant disparity between the frequency of HIDec and HIAcc.

Advances in technology now allow coaches and sports medical staff to track training intensity more precisely, helping to tailor conditioning needs, enhance performance and reduce injury risk [16, 17]. For instance, athletes who consistently train and perform near‐maximal intensity sprints (> 95% of their maximum velocity) and accumulate higher sprint volumes tend to have a lower risk of lower‐limb injuries compared to those who train at lower running intensities and maintain lower sprint volumes [18, 19]. However, assessing players solely through distance and speed variables may underestimate their actual external load [20]. This approach overlooks dynamic actions, specifically accelerations and decelerations, which have distinct biomechanical (kinetic, kinematic, spatiotemporal) and physiological (metabolic, neural, muscle‐tendon unit) demands on players [21]. A study by Dalen et al. [22], in elite Norwegian football, found that accelerations and decelerations contributed approximately 7% ± 10% and 5% ± 7% to the overall player load, respectively. These actions translated to 28% and 65% greater load per metre compared to other match‐day activities, highlighting the disproportionate physiological cost.

Consequently, HIAccs and HIDecs are now recognised as sensitive external workload markers of player stress [23]. Accelerations require substantial force and power, engaging fast‐twitch muscle fibres [24, 25]. In contrast, decelerations involve high eccentric forces that increase neuromuscular fatigue and the risk of tissue damage [21].

Although these patterns are well‐characterised in professional leagues, particularly in the English Premier League [26], semiprofessional football presents a distinct context. Players in Scottish semiprofessional teams typically train fewer times per week, have limited recovery resources, and face tactical, cultural and scheduling variations. These factors are likely to influence the magnitude and distribution of high‐intensity actions, yet these remain underexplored. Importantly, understanding these nuances can help align training and periodisation strategies with the true demands of semiprofessional match play.

Therefore, this study aimed to (1) quantify the total distance, frequency and peak magnitude of HIAcc and HIDec at two intensity thresholds (3‐4 m/s^2^ and > 4 m/s^2^) during competitive match play and training amongst semiprofessional Scottish football players and (2) examine positional differences in HIAcc and HIDec training load relative to competitive match play.

## 2. Methods

### 2.1. Design

A retrospective observational cohort study was conducted across the 2021/22 and 2022/23 competitive seasons of a male semiprofessional Scottish football club. GPS files from the team’s training sessions and matches were utilised to quantify acceleration and deceleration metrics. Position‐specific activities for match play and training sessions were established based on positional role and session type criteria. This study was designed and reported in accordance with the Strengthening the Reporting of Observational Studies in Epidemiology (STROBE) guidelines for cohort studies. Ethical approval was granted by the Divisional Research Ethics Committee (DivREC) for the Division of Dietetics, Physiotherapy, Podiatry and Radiography at Queen Margaret University on August 24, 2023. In line with University procedures at the time, individual reference numbers were not issued for divisional‐level approvals prior to September 2025. Consequently, no application reference number is available for this study, and a copy of the ethical approval release form is available on request. Consent from the club was obtained to use the data.

### 2.2. Participants

Player data for this study were obtained from thirty‐three semiprofessional male outfield football players from a club competing in the fourth tier of the Scottish Professional Football League. Height and body mass measurements were recorded monthly during both seasons. Data collection was a mandatory component of the players’ employment. Both players and club management granted consent for the use of training and match‐day data. Players were informed that de‐identified data collected as part of routine monitoring could be used for research purposes and were provided with the opportunity to opt out of data inclusion without consequence.

Participants’ characteristics were as follows (mean ± SD): age 25.33 ± 5.15 years, weight 80.12 ± 6.53 kg and height 181.90 ± 5.23 cm. Players were categorised by position: CDs (*n* = 8), FBs (*n* = 6), MF (*n* = 7), WMs (*n* = 5) and FWs (*n* = 7).

The team operated in a consistent 4–4–2 formation across both seasons, and all players retained their primary positional assignments throughout the study period. As is typical in competitive match play, natural tactical variations occurred within games, including shifts between defensive block heights, counter‐pressing sequences, counter‐attacking movements and brief positional cover when a teammate received treatment. These transient behaviours reflect normal fluid tactical adaptations rather than formal positional changes. Such variations are inherently present in football and are proportionally represented within the two‐season dataset, ensuring that positional classifications and workload interpretations remained valid.

### 2.3. Procedures

GPS technology was used to collect physical load data for all training sessions and match play during the 2021/22 and 2022/23 competitive seasons, excluding preseason. Training sessions occurred on outdoor synthetic grass surfaces and comprised integrated content including warm‐ups, technical drills, football conditioning games, small‐sided games, possession games, finishing drills and tactical exercises. Matches were played on outdoor grass or synthetic grass surfaces, adhering to Scottish Football Association rules [27]. Playing surface on match day was not systematically recorded across the dataset and therefore could not be modelled as a covariate. Similarly, although data were collected across two competitive seasons, season was not modelled as a separate covariate given the consistent tactical formation, staffing structure and playing squad retained throughout both seasons. Both factors are acknowledged as limitations of the retrospective study design.

Training data were investigated according to the number of days before a match (MD minus) [28]. A typical training week consisted of two sessions (MD‐4 and MD‐2) and one MD, with a 6‐day break between matches. MD + 1, MD + 2, MD‐3 and MD‐1 were designated as rest or recovery days and were excluded from analysis to maintain consistency.

Inclusion and exclusion criteria were established in line with previous research [29, 30]. A session‐level exclusion approach was applied. Only data from outfield players who were deemed fit (i.e., without injury preventing full participation) and who completed the entire team training session were included. For players who sustained injuries during the season, only data from specific training sessions or matches in which they were injured, unable to complete full participation, or otherwise unavailable were removed from the dataset.

The initial dataset contained 2339 individual observations. Following the application of predefined exclusion criteria—including the match exposure of fewer than 60 min, matches involving formation changes and incomplete observations due to injury, substitution or technical issues—1006 observations were excluded, resulting in 1333 observations retained for analysis. Per‐minute normalisation was considered but not adopted; only complete match observations were retained to ensure consistency across sessions, and per‐minute normalisation is considered less appropriate for discrete high‐intensity events such as accelerations and decelerations, where the frequency and distribution of efforts are not linearly proportional to playing time. Due to the retrospective nature of the dataset and overlap between exclusion criteria (e.g., substitutions resulting in fewer than 60 min of exposure and incomplete participation), mutually exclusive counts for each exclusion category were not retained. All exclusions were applied systematically and were not isolated to specific positional groups or session types, minimising the risk of systematic exclusion bias. Matches involving formation changes were excluded to preserve positional validity, as formation changes can substantially alter positional responsibilities and locomotor demands, thereby confounding position‐specific analysis.

### 2.4. External‐Load Variables and Data Collection

Player activity profiles were tracked using 10‐Hz GPS devices (PlayerTek Pod, 42 g, 84 × 42 × 21 mm; Catapult PlayerTek; Northern Ireland). Such systems have demonstrated acceptable reliability (coefficient of variation: 1.3%–5.9%) and validity (bias: < 5%) in measuring instantaneous velocity and acceleration in team sports contexts [31]. To ensure satellite communication, GPS devices were activated 15 min prior to warm‐up. Each participant consistently used the same unit to avoid interunit errors.

The physical parameters analysed included the following:

1. Number of accelerations (AccN) and number of decelerations (DecN)
2. Distance of accelerations (AccD) and distance of decelerations (DecD)
3. Peak acceleration (AccP) and peak deceleration (DecP)

Accelerations and decelerations were classified at thresholds of 3‐4 m/s^2^ and > 4 m/s^2^. Events were initially detected when acceleration exceeded 1 m/s^2^ and were counted only when this value remained above the relevant threshold for ≥ 1 s to minimise noise‐related artefacts. This minimum‐duration requirement (dwell time) ensured that only meaningful locomotive efforts were captured rather than brief fluctuations associated with GPS sampling variability. These thresholds align with Catapult’s validated acceleration framework [32] and are commonly used in elite football research [33]. Thresholds > 3 m/s^2^ are widely recognised as high‐intensity external load [34], whilst values > 4 m/s^2^ denote very high or near‐maximal efforts. Although no universal consensus exists, thresholds ≥ 3 m/s^2^ remain amongst the most frequently adopted in applied football monitoring [35].

Acceleration and deceleration magnitudes were derived from the rate of change in instantaneous velocity recorded at 10 Hz [36]. Importantly, efforts were identified based on the changes in velocity rather than absolute speed; therefore, players did not need to reach specific speed zones for an effort to be recorded [33]. AccP and DecP were defined as the highest instantaneous values recorded by the 10‐Hz GPS unit within each session and did not require a minimum duration [33, 35]. As such, peak metrics reflect isolated maximal locomotive efforts rather than sustained high‐intensity loading [33, 35].

Acceleration and deceleration distance metrics were included to quantify the cumulative mechanical demands associated with sustaining high‐intensity changes in velocity [35]. Sustained accelerations over greater distances generate higher total impulse, greater horizontal force production and increased neuromuscular and metabolic cost, whilst longer decelerations reflect prolonged eccentric‐braking exposure, which is strongly linked to mechanical fatigue and tissue loading [21, 37]. Repeated short‐distance accelerations and decelerations can also accumulate substantial mechanical stress despite minimal displacement [21, 38]. Therefore, distance reflects both the persistence and extent of mechanical loading and complements event frequency by capturing the cumulative mechanical work performed during high‐intensity locomotive actions [35, 38].

### 2.5. Statistical Analysis

Two‐way linear mixed models were employed using SPSS v28.0.0.0 (190) to properly account for the within‐subject correlation arising from repeated measures on the same players. The model included fixed effects for playing position (5 levels: CD, FB, MF, WM and FW) and session type (3 levels: MD, MD‐2 and MD‐4), with player as a random effect to account for individual variation. This approach addresses the limitation of one‐way ANOVA, which does not account for within‐subject correlation and can result in biased standard errors and *p* values. The alpha level was set at 0.05.

Pairwise comparisons were conducted with Bonferroni correction to reduce the likelihood of Type I errors (adjusted *α* = 0.0167 for three comparisons). Relative training load percentages concerning MD were determined using: (training metric/MD metric) × 100 [39]. This approach allowed for comprehensive assessment of training load relative to MD, providing insights into players’ preparation and performance.

Residual diagnostics were conducted for all count variables (AccN and DecN at both thresholds) by saving model residuals and examining normality via histograms, Q–Q plots and Shapiro–Wilk tests, alongside residual‐versus‐fitted scatterplots to assess homoscedasticity. The marginal *R* ^2^ for fixed effects (position and session) was extracted from each model as an index of explained variance.

## 3. Results

The initial dataset contained 2339 individual observations of in‐season MD (918) and training sessions (1,421) during the 2021/22 and 2022/23 seasons. Application of inclusion criteria resulted in 1333 individual observations (393 for MD and 940 for training sessions), with 1006 observations excluded based on predefined criteria as described in Section 2.3. Descriptive data (mean ± standard deviation) for acceleration and deceleration variables are provided for session type (Table 1) and position (Table 2).

**TABLE 1:** Descriptive statistics (mean ± standard deviation) for high‐intensity acceleration and deceleration metrics by session type.

Variable	Match day (*n* = 393)	MD‐2 (*n* = 501)	MD‐4 (*n* = 439)
*Acceleration metrics*			
AccD 3‐4 m/s^2^ (m)	50.38 ± 14.51	40.74 ± 12.36	53.54 ± 18.37
AccD > 4 m/s^2^ (m)	11.18 ± 4.71	10.37 ± 5.08	13.25 ± 7.79
AccN 3‐4 m/s^2^	59.67 ± 14.69	49.68 ± 12.21	68.13 ± 18.15
AccN > 4 m/s^2^	24.29 ± 8.36	22.26 ± 8.95	29.31 ± 13.32
Peak acceleration (m/s^2^)	6.00 ± 0.95	6.34 ± 0.88	5.67 ± 0.74

*Deceleration metrics*			
DecD 3‐4 m/s^2^ (m)	85.28 ± 24.50	47.63 ± 15.53	60.05 ± 24.08
DecD > 4 m/s^2^ (m)	38.40 ± 15.15	16.96 ± 8.80	21.24 ± 12.55
DecN 3‐4 m/s^2^	56.27 ± 13.99	42.30 ± 11.79	53.20 ± 18.42
DecN > 4 m/s^2^	35.89 ± 11.40	20.06 ± 8.13	25.18 ± 12.04
Peak deceleration (m/s^2^)	7.51 ± 1.33	6.89 ± 1.06	6.77 ± 1.45

*Note:* peak acceleration = AccP; peak deceleration = DecP; MD‐2 = training session 2 days before match; MD‐4 = training session 4 days before match.

Abbreviations: AccD = acceleration distance, AccN = acceleration number, DecD = deceleration distance, DecN = deceleration number, MD = match day.

**TABLE 2:** Descriptive statistics (mean ± standard deviation) for high‐intensity acceleration and deceleration metrics by playing position.

Variable	CD (*n* = 271)	FB (*n* = 247)	MF (*n* = 293)	WM (*n* = 196)	FW (*n* = 326)
*Acceleration metrics*					
AccD 3‐4 m/s^2^ (m)	41.74 ± 13.98	51.56 ± 18.93	47.69 ± 14.14	52.11 ± 15.54	47.48 ± 16.21
AccD > 4 m/s^2^ (m)	9.44 ± 4.86	11.74 ± 7.37	11.09 ± 4.80	14.79 ± 6.72	11.65 ± 5.97
AccN 3‐4 m/s^2^	55.69 ± 17.68	62.04 ± 17.69	61.32 ± 15.57	55.49 ± 16.45	58.25 ± 16.58
AccN > 4 m/s^2^	22.18 ± 9.77	25.77 ± 12.71	25.58 ± 9.79	28.63 ± 10.76	24.80 ± 10.55
Peak acceleration (m/s^2^)	6.09 ± 0.98	5.89 ± 0.92	5.95 ± 0.82	6.16 ± 0.88	6.04 ± 0.90

*Deceleration metrics*					
DecD 3‐4 m/s^2^ (m)	53.84 ± 19.05	72.17 ± 32.86	59.68 ± 22.01	62.85 ± 24.19	66.00 ± 28.18
DecD > 4 m/s^2^ (m)	19.75 ± 9.73	30.34 ± 19.05	22.84 ± 12.61	24.00 ± 13.90	26.61 ± 16.72
DecN 3‐4 m/s^2^	48.01 ± 14.71	53.29 ± 20.77	51.44 ± 14.33	46.17 ± 13.50	50.21 ± 15.42
DecN > 4 m/s^2^	23.65 ± 9.51	30.66 ± 16.20	26.56 ± 11.17	23.85 ± 9.90	26.89 ± 12.56
Peak Deceleration (m/s^2^)	7.10 ± 1.41	7.08 ± 1.27	6.99 ± 1.18	7.11 ± 1.38	6.95 ± 1.34

*Note:* MF = central midfielder; FW = forward.

Abbreviations: AccD = acceleration distance, AccN = acceleration number, DecD = deceleration distance, DecN = deceleration number, CD = central defender, FB = full back, WM = wide midfielder.

### 3.1. Main Effects of Position and Session

Linear mixed models revealed significant main effects for both position and session on all measured acceleration and deceleration variables (*p* < 0.001), apart from the position effect on peak deceleration (*p* = 0.456). No significant interaction between position and session was found for any acceleration or deceleration variable. To maintain conciseness, only statistically significant findings for the main effects are presented below.

The proportion of variance explained by the fixed effects of position and session (marginal *R* ^2^) ranged from 10.7% for AccN > 4 m/s^2^ to 30.7% for DecN > 4 m/s^2^, with intermediate values of 23.3% and 16.3% for AccN 3–4 m/s^2^ and DecN 3–4 m/s^2^, respectively. The remaining unexplained variance is consistent with field‐based sports science data where substantial between‐player variability attributable to individual physical capacity and tactical utilisation is anticipated.

### 3.2. Position‐Specific Differences in Match Play

Post hoc pairwise comparisons with Bonferroni correction revealed significant position‐specific differences across variables; key comparisons relevant to the study aims are summarised in Table 3. FB demonstrated significantly greater deceleration distances compared to CD, MF and WM across both intensity thresholds. WM showed significantly greater acceleration distances at > 4 m/s^2^ compared to CD, MF and FB.

**TABLE 3:** Significant pairwise comparisons by position.

Variable	Comparison	Mean difference	95% CI	*p*
Deceleration distance 3–4 m/s^2^	FB vs CD	16.645	(11.525, 21.764)	< 0.001
	FB vs MF	10.914	(5.887, 15.940)	< 0.001
	FB vs WM	7.024	(1.453, 12.595)	0.004
	FW vs CD	12.070	(7.290, 16.851)	< 0.001
	FW vs MF	6.339	(1.658, 11.021)	0.001
	WM vs CD	9.621	(4.166, 15.075)	< 0.001

Deceleration distance > 4 m/s^2^	FB vs CD	9.570	(6.658, 12.482)	< 0.001
	FB vs MF	6.544	(3.685, 9.403)	< 0.001
	FB vs WM	4.881	(1.712, 8.050)	< 0.001
	FW vs CD	6.794	(4.074, 9.513)	< 0.001

Deceleration number > 4 m/s^2^	FB vs CD	6.304	(3.749, 8.859)	< 0.001
	FB vs MF	3.440	(0.931, 5.949)	0.001
	FB vs WM	5.849	(3.069, 8.630)	< 0.001

Acceleration distance 3‐4 m/s^2^	WM vs CD	10.195	(6.293, 14.098)	< 0.001
	FB vs CD	9.584	(5.922, 13.247)	< 0.001
	FW vs CD	5.749	(2.329, 9.169)	< 0.001
	WM vs MF	4.301	(0.461, 8.141)	0.017
	FB vs MF	3.690	(0.093, 7.286)	0.040

Acceleration distance > 4 m/s^2^	WM vs CD	5.278	(3.742, 6.813)	< 0.001
	WM vs MF	3.637	(2.126, 5.148)	< 0.001
	WM vs FB	2.964	(1.396, 4.531)	< 0.001
	WM vs FW	3.058	(1.578, 4.538)	< 0.001
	FB vs CD	2.314	(0.873, 3.755)	< 0.001
	FW vs CD	2.220	(0.874, 3.565)	< 0.001
	MF vs CD	1.641	(0.261, 3.020)	0.008

*Note:* MF = central midfielder; FW = forward. Only statistically significant comparisons are presented. Mean differences with 95% confidence intervals and *p* values are shown following Bonferroni correction.

Abbreviations: AccD = acceleration distance, AccN = acceleration number, CD = central defender, DecD = deceleration distance, DecN = deceleration number, FB = full back, WM = wide midfielder.

### 3.3. Training vs Match‐Play Demands

**FIGURE 1:**
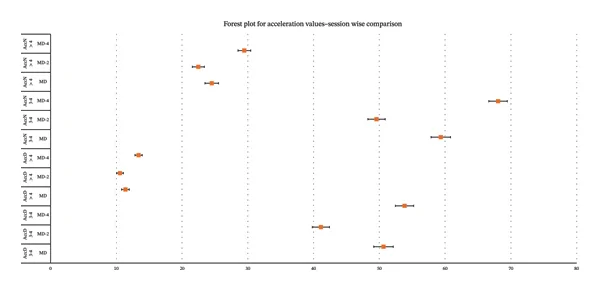
Forest plot for acceleration values—sessionwise comparison. Model‐based estimated marginal means with 95% confidence intervals for high‐intensity acceleration metrics across session types. Each variable (AccD 3‐4 m/s^2^, AccD > 4 m/s^2^, AccN 3‐4 m/s^2^, AccN > 4 m/s^2^) is displayed for match day (MD), MD‐2 and MD‐4 sessions. The *x* axis represents the estimated marginal mean value for each metric. Whiskers denote 95% confidence intervals derived from the linear mixed model. Note the consistently higher MD‐4 estimates relative to MD for all acceleration metrics, reflecting supramatch acceleration loading during the earlier training session. AccD = acceleration distance; AccN = acceleration number; MD = match day; MD‐2 = training 2 days before match; MD‐4 = training 4 days before match.

**FIGURE 2:**
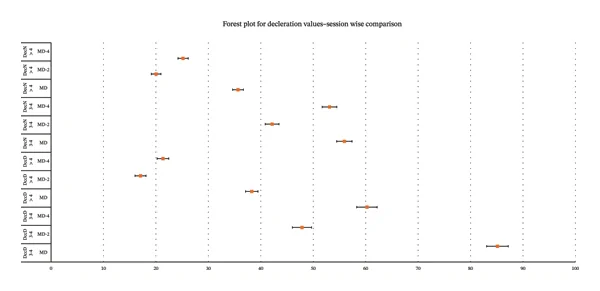
Forest plot for deceleration values—sessionwise comparison. Model‐based estimated marginal means with 95% confidence intervals for high‐intensity deceleration metrics across session types. Each variable (DecD 3‐4 m/s^2^, DecD > 4 m/s^2^, DecN 3‐4 m/s^2^, DecN > 4 m/s^2^) is displayed for match day (MD), MD‐2 and MD‐4 sessions. The *x* axis represents the estimated marginal mean value for each metric. Whiskers denote 95% confidence intervals derived from the linear mixed model. Match‐day estimates are consistently and substantially higher than both training sessions across all deceleration metrics, with MD‐2 demonstrating the lowest deceleration loads. DecD = deceleration distance; DecN = deceleration number; MD = match day; MD‐2 = training 2 days before match; MD‐4 = training 4 days before match.

Post hoc session comparisons revealed significant differences between match play and training days for all variables. Match days demonstrated significantly higher deceleration demands across all metrics compared to both training days. For acceleration metrics, MD‐4 showed higher values than both MD and MD‐2, particularly for AccN at both intensity thresholds. Figures 1 and 2 display the model‐based estimated marginal means with 95% confidence intervals for acceleration and deceleration metrics, respectively, across all three session types, visually illustrating these patterns, whilst Table 4 reports the corresponding pairwise mean differences, confidence intervals and *p* values.

**TABLE 4:** Session pairwise comparisons.

Variable	Comparison	Mean difference	95% CI	*p*
*Deceleration metrics*				
DecD 3‐4 m/s^2^	MD vs MD‐2	37.277	(33.933, 40.621)	< 0.001
	MD vs MD‐4	24.885	(21.438, 28.333)	< 0.001
	MD‐4 vs MD‐2	12.392	(9.150, 15.633)	< 0.001

DecD > 4 m/s^2^	MD vs MD‐2	21.207	(19.305, 23.110)	< 0.001
	MD vs MD‐4	16.932	(14.971, 18.893)	< 0.001
	MD‐4 vs MD‐2	4.275	(2.431, 6.119)	< 0.001

DecN 3‐4 m/s^2^	MD vs MD‐2	13.785	(11.401, 16.170)	< 0.001
	MD vs MD‐4	2.836	(0.378, 5.295)	0.017
	MD‐4 vs MD‐2	10.949	(8.637, 13.261)	< 0.001

DecN > 4 m/s^2^	MD vs MD‐2	15.630	(13.961, 17.299)	< 0.001
	MD vs MD‐4	10.482	(8.761, 12.202)	< 0.001
	MD‐4 vs MD‐2	5.149	(3.531, 6.766)	< 0.001

*Acceleration metrics*				
AccD 3‐4 m/s^2^	MD vs MD‐2	9.515	(7.123, 11.908)	< 0.001
	MD‐4 vs MD	3.203	(0.737, 5.669)	0.006
	MD‐4 vs MD‐2	12.719	(10.399, 15.038)	< 0.001

AccD > 4 m/s^2^	MD‐4 vs MD	1.989	(1.019, 2.959)	< 0.001
	MD‐4 vs MD‐2	2.820	(1.908, 3.732)	< 0.001

AccN 3‐4 m/s^2^	MD vs MD‐2	9.775	(7.367, 12.182)	< 0.001
	MD‐4 vs MD	8.703	(6.222, 11.185)	< 0.001
	MD‐4 vs MD‐2	18.478	(16.144, 20.812)	< 0.001

AccN > 4 m/s^2^	MD vs MD‐2	2.033	(0.370, 3.697)	0.010
	MD‐4 vs MD	4.959	(3.244, 6.674)	< 0.001
	MD‐4 vs MD‐2	6.992	(5.379, 8.605)	< 0.001

*Peak values*				
Peak deceleration	MD vs MD‐2	0.621	(0.415, 0.828)	< 0.001
	MD vs MD‐4	0.746	(0.533, 0.959)	< 0.001

Peak acceleration	MD‐2 vs MD	0.340	(0.201, 0.478)	< 0.001
	MD‐2 vs MD‐4	0.674	(0.539, 0.808)	< 0.001

*Note:* MD‐2 = training session 2 days before match; MD‐4 = training session 4 days before match. Only statistically significant comparisons are presented. Mean differences with 95% confidence intervals and *p* values are shown following Bonferroni correction.

Abbreviations: AccD = acceleration distance, AccN = acceleration number, DecD = deceleration distance, DecN = deceleration number, MD = match day .

### 3.4. Relative Training Load Analysis

Analysis of training loads relative to match play revealed distinct patterns across metrics (Table 5). During MD‐4, several acceleration metrics exceeded 100% of match‐play values: AccD 3‐4 m/s^2^ (106.3%), AccD > 4 m/s^2^ (117.4%), AccN 3‐4 m/s^2^ (114.7%) and AccN > 4 m/s^2^ (120.2%). In contrast, all deceleration metrics during training remained below match values, with MD‐2 showing the lowest relative loads (DecD 3‐4 m/s^2^ 56.2%, DecD > 4 m/s^2^ 44.6%, DecN 3‐4 m/s^2^ 75.4%, DecN > 4 m/s^2^ 56.2%).

**TABLE 5:** Training load as percentage of match‐day values by session, with 95% confidence intervals.

Variable	MD‐2 (%)	95% CI	MD‐4 (%)	95% CI
DecD 3‐4 m/s^2^	56.2	(53.7, 58.7)	70.8	(67.9, 73.6)
DecD > 4 m/s^2^	44.6	(41.5, 47.6)	55.7	(52.4, 59.1)
DecN 3‐4 m/s^2^	75.4	(72.3, 78.4)	94.9	(91.4, 98.4)
DecN > 4 m/s^2^	56.2	(53.1, 59.2)	70.6	(67.2, 74.0)
AccD 3‐4 m/s^2^	81.2	(77.7, 84.7)	106.3	(102.2, 110.5)
AccD > 4 m/s^2^	92.7	(86.2, 99.2)	117.5	(109.8, 125.1)
AccN 3‐4 m/s^2^	83.5	(80.5, 86.6)	114.7	(111.0, 118.4)
AccN > 4 m/s^2^	91.7	(86.4, 97.0)	120.2	(113.8, 126.6)

*Note:* Values > 100% indicate training loads exceeding match‐day demands. MD‐2 = training session 2 days before match; MD‐4 = training session 4 days before match. Values represent mean training loads relative to match‐day demands, calculated as (training metric/match‐day metric) × 100.

Abbreviations: AccD = acceleration distance, AccN = acceleration number, DecD = deceleration distance, DecN = deceleration number, MD = match day.

Position‐specific analysis revealed that FB demonstrated the most pronounced differences between training and match play, particularly for deceleration metrics, where MD‐2 values were only 56% and 75% of match values for DecD and DecN, respectively. WM showed the highest relative acceleration loads during MD‐4, exceeding match values by up to 124% for AccN 3‐4 m/s^2^.

## 4. Discussion

This study provides novel insights into the external acceleration and deceleration load demands of semiprofessional Scottish footballers, revealing distinct patterns that differ from those reported in professional football. The use of linear mixed models allowed for robust analysis accounting for the repeated‐measures nature of the data.

### 4.1. Position‐Specific Demands

Analysis showed that playing position substantially influences physical demands in semiprofessional football. The FB position emerged as having the highest overall external load, particularly for deceleration metrics. Significant differences between FB and CD in deceleration distance (16.65 m at 3–4 m/s^2^; 9.57 m at > 4 m/s^2^) and frequency reflect the evolving demands of modern FB roles [40].

Modern FBs are required to engage between offensive and defensive phases, executing high‐intensity attacking play (overlapping and underlapping actions) when in possession, and rapid HIDec within confined spaces to fulfil defensive requirements. This may contribute to the significantly higher HIDec observed in FB compared to central positions. These findings align with Tierney et al. [15], who indicated that players in lateral positions exhibit the most significant disparities in the frequency of HIDec compared to HIAcc.

WM demonstrated significantly greater acceleration distances than CD and MF, consistent with their tactical responsibility in exploiting wide spaces and creating attacking opportunities [41]. The 5.28 m (mean difference) greater AccD > 4 m/s^2^ for WM compared to CD represents a difference that should be considered with respect to positional demands. Notably, all outfield positions showed significantly greater acceleration distances than CD at the > 4 m/s^2^ threshold (FW: +2.22 m, FB: +2.31 m, MF: +1.64 m, WM: +5.28 m), demonstrating that CDs have distinctly lower high‐intensity acceleration demands. At the 3‐4 m/s^2^ threshold, WM also exceeded MF (+4.30 m) and FB exceeded MF (+3.69 m), indicating a positional hierarchy where wide positions (WM, FB) demonstrate greater acceleration demands than central positions (CD, MF). These positional differences are consistent with previous work reporting superior acceleration and deceleration densities in wide positions, although the specific magnitudes differ in this semiprofessional context [14, 42].

#### 4.1.1. Central Positional Constraints, Structure and Team Tactics

Central positions (CD and MF) also showed lower HIAcc and HIDec values overall, which may be attributed to multiple interrelated factors beyond tactical labelling alone. Spatial constraints in central areas and the greater defensive responsibilities typically assigned to central players likely reduce opportunities for repeated high‐intensity accelerations and decelerations, as central zones are more congested and often involve more position‐specific defensive organisation [7, 9]. These lower values may also reflect positional and individual characteristics, such as the larger/heavier morphology often observed in CDs, increased spatial congestion and opposition pressure in central zones, and differences in fitness levels and movement preferences [9, 42]. Tactical context undoubtedly influences these patterns, as match context and tactical decisions directly affect player movement demands [43]. However, players’ tactical functions are inherently dependent on team philosophy and tactical approaches [44], which are themselves dynamic rather than fixed. Over the two‐season study period, it is likely that tactical nuances, if not fundamental approaches, evolved in response to opposition analysis, player availability and strategic development. Therefore, whilst these findings reflect the tactical approaches employed during the study period, the observed positional patterns may also represent more stable structural constraints inherent to football positions and distinguishing between tactical and structural influences remains methodologically challenging.

#### 4.1.2. Contextual Determinants of Peak Deceleration

In contrast to these positional differences in deceleration frequency and distance metrics, peak deceleration magnitude did not differ significantly between positions (*p* = 0.456), with peak values of approximately 7.0 m/s^2^ observed across roles. This finding is notable given the well‐established literature demonstrating clear positional patterns in acceleration and deceleration volume. Delaney et al. [45] reported that CDs and FWs performed fewer accelerations and decelerations than wide defenders during the most demanding passages of play, whereas wide defenders demonstrated the greatest acceleration and deceleration demands. These patterns align closely with our significant findings for deceleration frequency and distance metrics, making the absence of positional differences in peak deceleration more striking. Similarly, Morgans et al. [46] observed significant positional differences in deceleration volumes, but nonsignificant differences in peak decelerations for certain attacking roles. The consistent evidence suggests that whilst tactical demands strongly influence deceleration frequency and volume, maximum deceleration capacity may be more individually determined than positionally dependent. Peak deceleration likely represents physiological and mechanical capacity constraints that are more individually variable than volume‐based metrics, which primarily reflect tactical requirements and spatial responsibilities.

Beyond these positional and individual considerations, contextual factors further help to explain the absence of positional differences in peak deceleration. Contextual analyses from elite competitions have shown that acceleration–deceleration exposure is heavily modulated by ball‐possession status, phase of play and opponent ranking, with similar positional labels demonstrating different high‐intensity profiles under different tactical game models [46, 47]. Complementary work examining sprinting from a tactical perspective indicates that maximal or near‐maximal sprint actions typically arise from specific scenarios such as counterattacks, recovery runs and pressing triggers rather than fixed positional templates [11]. Studies of match peak speeds, maximum accelerations and maximum decelerations further show that the expression of maximal locomotor capacities is highly dependent on game state, defensive organisation and transition moments [48]. Taken together, these findings support the interpretation that positional role primarily constrains the frequency and volume of acceleration–deceleration load, whereas peak values are more episodic, individually constrained and context‐dependent and therefore less likely to follow neat positional hierarchies.

#### 4.1.3. Interindividual Variability and Player‐Specific Profiles

Substantial interindividual variability further contributes to these patterns. Even within the same positional group, players can differ markedly in maximal sprint speed, sprint mechanical profile, braking capacity and neuromuscular characteristics. Recent in situ acceleration–speed profiling has demonstrated large between‐player differences in theoretical maximal acceleration and speed within the same squad and positional line, despite similar positional labels [49]. Narrative and review work on positional profiles similarly highlights that playing position interacts with, rather than determines, individual locomotor characteristics, reinforcing the value of individualised profiling within positional categories [42]. In addition, studies employing individually derived high‐intensity thresholds for accelerations and decelerations have shown that players occupying similar nominal positions can display markedly different high‐intensity exposure profiles across a season, depending on their individual capacities and tactical utilisation within the team structure [50]. The marginal *R* ^2^ values of 10.7%–30.7% observed in the present study are consistent with this interpretation, indicating that whilst position and session type account for a meaningful proportion of variance, the majority reflects between‐player differences in physical capacity and contextual factors. Collectively, this evidence supports the view that positional groupings provide useful but coarse descriptors of external load and that substantial between‐player variability should be anticipated even within the same role.

#### 4.1.4. Interpretation of Positional Profiles

In the context of the present results, this combination of positional role, match context, structural and spatial constraints, evolving team tactics and interindividual variability helps to explain why AccP and DecP values did not mirror the clear positional differences observed for volume‐based metrics. Practically, the current positional profiles should therefore be interpreted as broad descriptors of role‐specific external load that sit on top of meaningful between‐player and between‐team variations driven by physical capacity, tactical game model and situational match demands, rather than as rigid templates that apply uniformly to all players within a given position.

### 4.2. Training Periodisation in Semiprofessional Football

The results offer insights into training periodisation in semiprofessional football, where schedules differ markedly from professional contexts [51]. Acceleration metrics during MD‐4 frequently exceeded match‐day values (106%–120%), with AccD 3–4 m/s^2^ at 106% and AccN > 4 m/s^2^ reaching 120%, contrasting sharply with professional studies reporting submaximal MD‐4 loads [30, 52]. This elevated MD‐4 acceleration stimulus likely reflects a deliberate compensatory strategy to maintain HIAcc adaptations within the constraints of only two weekly training sessions. This aligns with Clemente et al. [53] and Baptista et al. [54], who observed that both HIAcc and HIDec during training can closely resemble or exceed match values when overall weekly training volume is limited, reflecting a periodised approach to stimulus distribution.

In contrast, all deceleration metrics during training were substantially below match values, with MD‐2 particularly underloading players relative to competition (DecD 3‐4 m/s^2^ = 56%, DecD > 4 m/s^2^ = 45%, DecN 3–4 m/s^2^ = 75%, DecN > 4 m/s^2^ = 56%). This pattern may be intentionally structured as a lower‐intensity session within a periodised tapering framework, designed to manage residual fatigue, preserve neuromuscular readiness and optimise preparedness for match play. By reducing high‐intensity acceleration and deceleration exposure on MD‐2, players are better positioned to tolerate the principal high‐intensity stimulus delivered on MD‐4. Such scheduling balances the maintenance of training adaptations with optimal physiological freshness for competitive performance.

This asymmetry between acceleration and deceleration exposure is particularly important. High‐intensity decelerations impose greater eccentric forces, neuromuscular fatigue and injury risk than accelerations [21]. The higher frequency of HIDec compared to HIAcc during matches (DecN > 4 m/s^2^: 35.7 vs AccN > 4 m/s^2^: 24.5) corroborates previous research [13, 55]. Although MD‐4 provides an adequate or even supramatch acceleration stimulus, current training does not sufficiently replicate the high deceleration demands encountered during competitive matches, potentially leaving players underprepared for match‐specific eccentric loading.

Collectively, these findings suggest that semiprofessional teams may inadvertently prioritise acceleration over deceleration development. Periodisation strategies should therefore retain MD‐4 as the principal high‐intensity loading day, whereas MD‐2 is deliberately structured as a lower‐intensity session to manage residual fatigue, maintain fitness adaptations and optimise neuromuscular readiness for match play. Additionally, incorporating targeted deceleration drills such as reactive stopping, change‐of‐direction under load and defensive small‐sided games into MD‐4 sessions could better replicate match demands, enhance player preparedness and reduce injury risk.

### 4.3. Practical Applications

#### 4.3.1. Mechanical and Neuromuscular Demands of High‐Intensity Deceleration

The positional acceleration–deceleration profiles observed in this study have clear implications for strength and conditioning practice. High‐intensity decelerations are not only frequent in semiprofessional football but are also mechanically demanding actions that impose substantial eccentric load on the lower‐limb musculature. Biomechanical evidence indicates that rapid horizontal decelerations are characterised by high braking impulses and a ground reaction force profile with pronounced impact peaks and loading rates, reflecting the unique mechanical demands of reducing whole‐body momentum over short timeframes [21, 38, 56]. These stresses place considerable demands on the lower‐limb musculature to dissipate braking loads and maintain joint stability, and repeated exposure can contribute to tissue damage, neuromuscular fatigue and longer‐term mechanical fatigue failure processes if not appropriately managed [21, 38], reinforcing the need for targeted preparation of these demands in training.

#### 4.3.2. Eccentric Strength, Horizontal Braking Capacity and Change‐of‐Direction Performance

In the present study, FBs and WMs exhibited the greatest high‐intensity deceleration distances, consistent with their frequent involvement in high‐speed advances followed by abrupt braking when transitioning between attack and defence. From a strength and conditioning perspective, these positional profiles suggest that wide players may require enhanced eccentric‐braking capacity and progressive exposure to high‐intensity deceleration to meet match‐specific demands. Cross‐sectional evidence in footballers shows that greater eccentric and concentric knee extensor and flexor strength is associated with superior maximal linear deceleration ability and shorter stopping distances [37, 56]. Work on change‐of‐direction mechanics further demonstrates that athletes who generate high horizontally directed braking and propulsive forces in the penultimate and final foot contacts, whilst maintaining appropriate trunk and lower‐limb alignment, achieve superior change‐of‐direction performance. More broadly, braking and change‐of‐direction performance are supported by the ability to generate and tolerate high horizontal braking forces and to execute effective multistep braking strategies under higher approach velocities, reinforcing the importance of braking‐specific strength and technical development within football conditioning [38].

#### 4.3.3. Training Interventions Targeting Eccentric Braking and Change‐of‐Direction Ability

Intervention studies and systematic reviews highlight that eccentric‐braking qualities are trainable and respond well to targeted programming. Eccentric‐overload exercises such as flywheel squats, lunges and Romanian deadlifts have been shown to improve change‐of‐direction speed and braking kinetics in footballers by increasing eccentric strength and braking impulse [57, 58]. Recent meta‐analytic and conceptual work indicates that training programmes that explicitly enforce high‐velocity braking emphasise eccentric and reactive strength and progressively increase approach velocity and braking angle are more effective for improving deceleration performance than traditional strength or small‐sided games alone [21]. For practitioners, this means that alongside conventional strength and power training, deceleration‐ and change‐of‐direction‐specific drills should be systematically progressed and individualised, particularly for positions with higher match‐day deceleration exposure.

#### 4.3.4. Monitoring, Load Prescription and Position‐Specific Profiling

The intensity of accelerations and decelerations should be considered when designing and monitoring training. Manipulating acceleration intensity in repeated‐sprint drills substantially increases both external mechanical stress and internal responses such as heart rate and perceived exertion, even when distance and peak speed are matched [59]. Research on mechanical and metabolic cost further shows that deceleration and change‐of‐direction phases may present relatively low estimated metabolic power despite high muscular and eccentric demand, meaning traditional metabolic or distance‐based measures risk underestimating true mechanical load [60, 61]. These findings support the use of acceleration–deceleration metrics, including high‐intensity distance alongside conventional training load measures when prescribing and monitoring work.

The positional profiles reported in this study can therefore be used as benchmarks for designing individualised, position‐specific strength and conditioning programmes. This includes determining appropriate frequencies and distances of high‐intensity accelerations and decelerations, identifying players who may require additional eccentric‐braking preparation and ensuring that mechanical fatigue‐related injury risk is mitigated across the competitive season.

### 4.4. Limitations

Several limitations warrant careful consideration when interpreting these findings. First, only players who completed entire training sessions were included, which excluded sessions in which players were managing minor injuries or fatigue or were in later stages of return‐to‐play. Whilst this improved consistency and reduced noise, it may limit generalisability to applied settings where modified participation is common and where acceleration–deceleration behaviour may differ under load restriction.

The study examined a single semiprofessional club competing in the fourth tier of Scottish football. Although the mixed‐model structure partially accounts for repeated measures and between‐player variability, club‐specific factors (e.g., coaching approach, weekly scheduling constraints, pitch type/conditions and opponent style) may have influenced the observed positional profiles. The consistent 4–4–2 structure across the study period supports internal consistency, but positional demands may differ under alternative tactical systems and competitive contexts. Playing surface on match day was not systematically recorded and could therefore not be controlled as a covariate. Similarly, whilst data were collected across two seasons within a consistent tactical and staffing structure, season was not modelled as a separate variable. These uncontrolled factors represent a limitation of the retrospective study design and should be addressed in future prospective work. As a retrospective census of a single squad, no prospective sample‐size estimation was undertaken; nonetheless, the two‐season observation window and the number of retained observations (*n* = 1333) meet or exceed those of comparable positional acceleration–deceleration studies, most of which are based on a single competitive season [14, 30, 53].

Acceleration and deceleration count variables (AccN and DecN) were modelled using linear mixed models. Residual diagnostics revealed near‐normal distributions for acceleration count residuals, whilst deceleration count residuals showed more substantial positive skew and kurtosis (skewness = 1.95 and 1.87; kurtosis = 27.39 and 20.36 for DecN 3–4 and > 4 m/s^2^, respectively). However, model‐estimated and observed residual variances corresponded closely across all variables, and marginal *R* ^2^ values of 10.7%–30.7% are consistent with field‐based data where interindividual variability accounts for a meaningful proportion of unexplained variance. Whilst the large sample size and consistently large F‐statistics support the robustness of the conclusions, future work should consider generalised linear mixed models with negative binomial distributions for count outcomes in this context.

Important methodological constraints relate to the GPS/GNSS technology used. Whilst 10‐Hz units typically demonstrate acceptable validity and reliability for total distance and average speed [31, 62, 63], measurement quality declines when estimating instantaneous velocity during high acceleration and braking actions [64, 65]. Acceleration and deceleration metrics are also sensitive to manufacturer algorithms, filtering and event‐definition criteria [2, 66], and therefore, absolute magnitudes should be interpreted cautiously.

The study used generic absolute acceleration thresholds (≥ 3 and > 4 m/s^2^). Emerging evidence indicates that absolute thresholds can misrepresent high‐intensity exposure when players differ substantially in maximal acceleration/deceleration capacity. Individualised profiling or relative thresholds may provide a more precise representation of player‐specific mechanical load [55].

Finally, GPS‐derived external‐load metrics cannot directly quantify the metabolic, neuromuscular or perceptual cost of acceleration–deceleration actions. The absence of injury surveillance, wellness and fatigue measures also prevents any direct assessment of whether the observed training–match deceleration imbalance translated into increased fatigue or injury risk. Future research should integrate internal‐load measures, neuromuscular and perceptual fatigue monitoring, and injury surveillance alongside individual profiling to better link acceleration–deceleration exposure to health and performance outcomes.

## 5. Conclusion

This study advances understanding of external load in semiprofessional football, demonstrating distinct patterns from professional contexts. The use of linear mixed models confirmed significant effects of both playing position and training/match timing on acceleration and deceleration demands. Key findings include the elevated deceleration requirements of FBs, the higher acceleration demands of WMs and a unique periodisation pattern in which training acceleration loads exceeded match values, whereas deceleration remained under‐represented.

These findings have important implications for practice. The limited training time in semiprofessional football requires careful load management to balance adequate stimulus with recovery. The observed acceleration–deceleration asymmetry in training suggests a need for greater emphasis on deceleration preparation, particularly for positions with high match demands. Position‐specific training approaches are essential to address the varied mechanical and tactical demands across playing roles.

Importantly, the positional acceleration–deceleration profiles identified in this study also provide a foundation for developing individualised, role‐specific training programmes. Aligning eccentric strength, deceleration and change‐of‐direction training to the mechanical demands experienced by each position may enhance performance in high‐intensity transitions whilst helping to manage cumulative braking loads across the season.

Future research should examine these patterns across multiple teams and leagues to determine whether these findings reflect broader trends in semiprofessional football or are specific to the studied context. Additionally, integrating injury incidence and internal‐load monitoring with acceleration–deceleration exposure would provide valuable insight into the relationship between mechanical load, performance and injury risk in semiprofessional players.

## Funding

This research received no specific grant or funding from any funding agency in the public, commercial or not‐for‐profit sectors.

## Conflicts of Interest

The authors declare no conflicts of interest.

## Data Availability

The datasets generated and analysed during the current study are not publicly available due to confidentiality agreements with the participating football club but are available from the corresponding author on reasonable request.
